# Global Cholera Epidemiology: Opportunities to Reduce the Burden of Cholera by 2030

**DOI:** 10.1093/infdis/jiy486

**Published:** 2018-09-01

**Authors:** Dominique Legros

**Affiliations:** World Health Organization, Geneva, Switzerland

## Abstract

While safe drinking water and advanced sanitation systems have made the Global North cholera-free for decades, the disease still affects 47 countries across the globe resulting in an estimated 2.86 million cases and 95,000 deaths per year worldwide. Cholera impacts communities already burdened by conflict, lack of infrastructure, poor health systems, and malnutrition. In October 2017, the Global Task Force on Cholera Control (GTFCC) launched an initiative titled Ending Cholera: A Global Roadmap to 2030, with the objective to reduce cholera deaths by 90% worldwide, and eliminate cholera in at least 20 countries by 2030. The GTFCC is working to position cholera control not as a vertical programme but instead using cholera as a marker of inequity and an indicator of poverty, linking the objectives of the Roadmap to the SDGs. The roadmap consists of targeted multi-sectoral interventions, supported by a coordination mechanism, along 3 axes: (1) early detection and quick response to contain outbreaks; (2) a multisectoral approach to prevent cholera recurrence in hotspots; (3) an effective partnership mechanism of coordination for technical support, countries capacity building, research and M&E, advocacy and resource mobilization. Every case and every death from cholera is preventable with the tools we have today.

Cholera is a disease of inequity, an ancient illness that today sickens and kills only the world’s poorest and most vulnerable persons. Cholera infection occurs when high concentrations of *Vibrio cholerae* are ingested via fecally contaminated food or water. A severe case causes rapid dehydration and can kill a person within hours. Uncontrolled cholera outbreaks, endemicity, and repeated outbreaks are devastating to communities and to their prospects for development. Although it was eliminated from the Global North >150 years ago, cholera still kills an estimated 95000 persons per year and sickens 2.86 million more [1]. Cholera infections do not occur by chance; rather, cholera affects communities already burdened by conflict, lack of infrastructure, poor health systems, and malnutrition. In fact, the map of cholera outbreaks is essentially the same as a map of poverty and marginalization.

Now more than ever, we are in a position to fundamentally change this map by using improved data on cholera cases and deaths to strategically target a multisectoral approach that includes essential water, sanitation, and hygiene (WASH) services. Using such an approach, it is feasible not only to significantly reduce the burden of cholera but also to improve equity and put countries on the right trajectory for meeting the targets of the sustainable development goals (SDGs). This multisectoral approach to cholera control is captured in the renewed global strategy for cholera control, outlined in *Ending Cholera: A Global Roadmap to 2030* [2]. This effort of the partners of the Global Task Force on Cholera Control (GTFCC) calls for focusing on the 47 countries affected by cholera today, supporting countries to develop comprehensive, multisectoral cholera control plans.

The *Global Roadmap* targets a 90% reduction in cholera deaths by 2030. During this period, the GTFCC estimates that as many as 20 countries could eliminate cholera. (A country will be considered to have eliminated cholera when it reports no confirmed cases with evidence of local transmission for ≥3 consecutive years and has a well-functioning epidemiologic and laboratory surveillance system able to detect and confirm cases.) The *Global Roadmap* will encourage a phased approach that takes into consideration the current conditions on the ground, helping each country set a realistic and achievable goal for cholera control.

The renewed strategy provides an effective mechanism for synchronizing the efforts of countries, donors, and technical partners, allowing for a coordinated, multisectoral support to country-level implementation of cholera control measures. It has 3 axes: (1) a focus on cholera hot spots (defined as areas affected by recurrent outbreaks of cholera that tend to occur year after year, often during the rainy season) in endemic countries; (2) early detection and response to contain epidemics quickly; and (3) an effective mechanism of coordination for technical support and resources at global and country levels. Critical to this effort will be high-quality data, which are needed for rapid detection and confirmation of outbreaks so that they can be contained to precisely identify cholera hot spots for well-targeted interventions and to monitor progress and adjust public health control measures in all settings.

## IMPROVING DATA ON DISEASE BURDEN

The overall global burden of cholera is poorly understood because only a small fraction of cases are reported to the World Health Organization (WHO). Underreporting occurs mostly because of weak or absent surveillance systems, although countries also face disincentives for reporting cases, such as risks to tourism and export industries.

Models used to estimate cholera burden are helpful for understanding the overall burden of the disease and its cost to society, but controlling cholera requires detailed, accurate data based on enhanced epidemiologic and laboratory surveillance systems that can identify hot spots and detect and confirm outbreaks early to facilitate a quick response. Without these systems in place, governments and cholera control partners are forced to make critical public health decisions based on limited data. Improving data quality, beginning with outbreak detection and confirmation and carrying through to robust country reporting, must be of highest priority in the fight against cholera.

**Figure 1.**
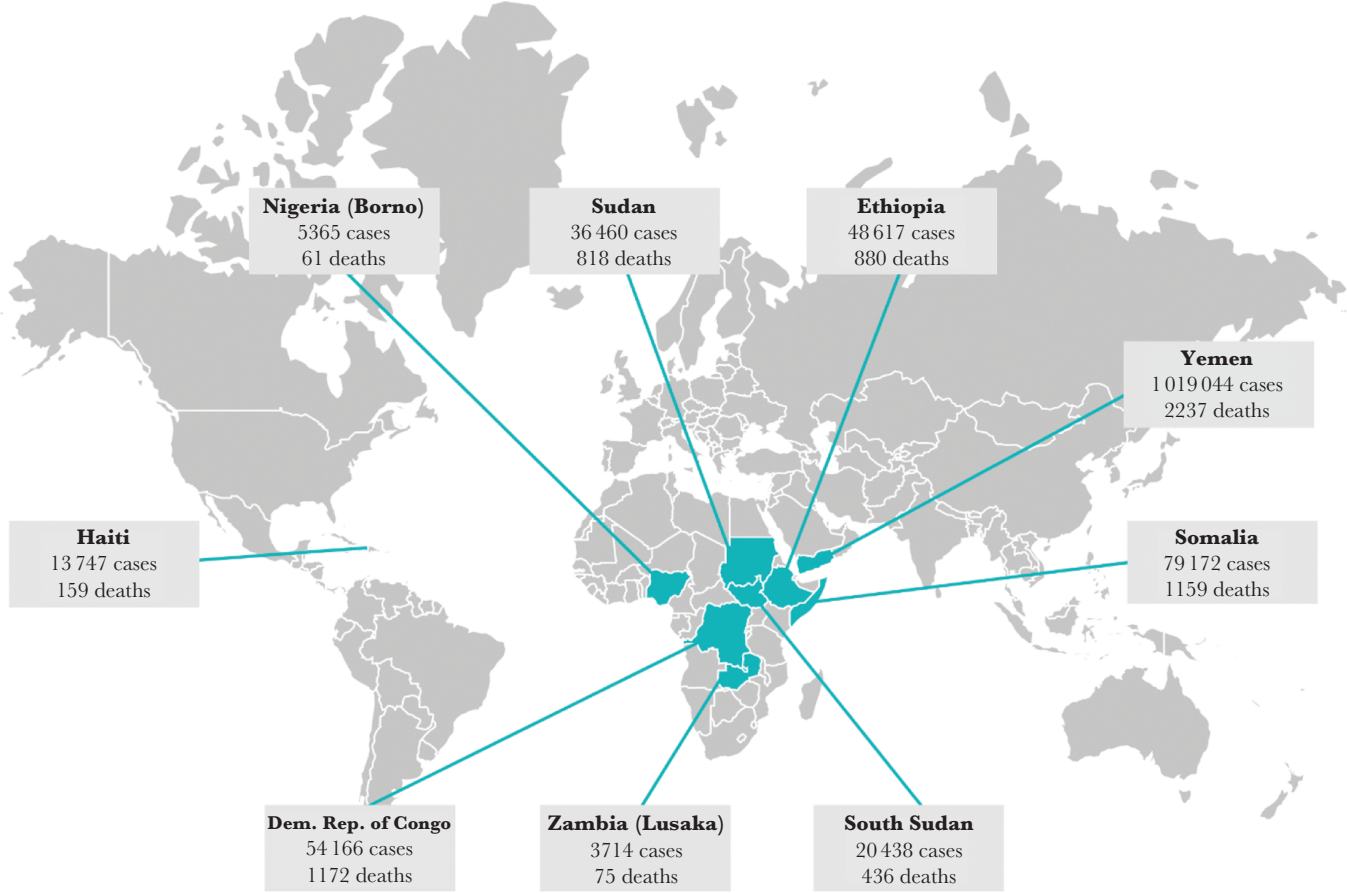
Major cholera outbreaks in 2017 (source: World Health Organization).

What we do know based on available data is that 2017 was an especially bad year for cholera. Forty-seven countries are affected by cholera today, and 9 countries had large outbreaks in 2017, as shown in Figure 1 [3]. In addition to Yemen, explosive outbreaks spread in the Democratic Republic of Congo, Ethiopia, Haiti, Nigeria (Borno), Somalia, South Sudan, Sudan, and Zambia (Lusaka) during 2017. It is far from clear, however, that 2017 will ultimately prove to be an outlier. Conflict, climate change, urbanization, and population growth are all serving to increase the risk of severe cholera outbreaks, and the annual cholera burden will continue to rise unless countries act to implement multisectoral cholera control plans in regions at risk.

## CHOLERA CONTROL AS PATHWAY TO IMPROVED EQUITY

Considering that cholera outbreaks and deaths are completely preventable with the technology and tools we have today, the continued existence of cholera outbreaks is a violation of human rights and dignity. However, there are also important strategic reasons to target cholera: it is a highly sensitive, highly specific indicator for extreme poverty and harsh living conditions. The presence of cholera indicates that a population does not have access to even basic water or basic sanitation, the first rungs on the WASH development ladder, and thus identifies areas in urgent need of WASH investment. (A basic water supply is defined as access to safe drinking water sources—household connection, public standpipe, borehole, protected dug well, protected spring, or rainwater collection—within a 30-minute round trip, plus household or other disinfection, and basic sanitation is defined as access to improved sanitation facilities, including connection to a public sewer or to a septic system, or a pour-flush, simple pit, or ventilated improved pit latrine.) Cholera hot spots can help us identify and target the communities that are being left behind in the effort to achieve the SDGs. Because hot spots play an important role in the spread of the disease to other regions, controlling cholera in hot spots is also a critical part of the strategy to end cholera globally.

A recent effort conducted by the GTFCC partners as part of the renewed global strategy for cholera control, the *Global Roadmap,* mapped the burden of cholera in sub-Saharan Africa in cholera hot spots—areas of endemicity characterized by weak infrastructure and recurrent outbreaks. The mapping showed that 4% of the African population live in the areas most at risk for cholera and, therefore, in priority areas to target for WASH investment. Targeting the districts with the highest incidence first—districts with an estimated population of 35 million inhabitants, or 4% of the population— could eliminate 50% of the region’s cholera burden [4].

Even though WASH services have been recognized by the United Nations as a human right, >2 billion persons worldwide drink water from sources that are fecally contaminated [5]. If we focus on providing safe water and sanitation facilities and changing hygiene behavior among the world’s most vulnerable persons, it will help countries reach the SDGs, yielding progress against goals such as SDG 2 (“End hunger and improve nutrition”), SDG 3 (“Good health and well-being”), SDG 6 (“Clean water and sanitation”), and SDG 10 (“Reduced inequalities”). If, on the other hand, we fail to provide minimum WASH services, particularly to the most vulnerable populations, the SDGs become unattainable. Lack of access to WASH services is the main reason that diarrheal diseases remain the second-largest killer of children worldwide [6].

## *GLOBAL ROADMAP* AS OPPORTUNITY TO CONTROL CHOLERA

A number of factors have converged in recent years to make cholera elimination an actionable and feasible objective. First, the heightened emphasis on equity and “reaching the last mile,” championed through the SDGs, elevates the importance of addressing cholera and other diseases of extreme poverty. Cholera incidence likewise offers a valuable indicator of the effectiveness of WASH interventions among these vulnerable populations.

Second, we have the technical capacity to detect cholera quickly so that we can respond immediately, preventing large-scale, uncontrolled outbreaks. Positive stool culture or polymerase chain reaction tests are the reference standard for confirmation of cholera, but in regions where laboratory capacity is limited, rapid diagnostic tests can now be used to test for suspected cholera when patients present clinically with acute watery diarrhea. This allows governments to initiate a rapid response to cholera while awaiting confirmation of the diagnosis via stool culture or polymerase chain reaction.

Third, we now have oral cholera vaccine (OCV) in sufficient quantity to fight cholera on a large scale. There are 3 World Health Organization–prequalified OCVs, which are safe, inexpensive, easy to deliver, and effective. A person can be fully vaccinated for just $6 [7]. OCV is a game-changer because it takes effect immediately and, with 2 doses, works to prevent cholera for 2–3 years, effectively bridging the emergency response period with a long-term development approach. In addition to immediately averting loss of life in emergency situations, it provides governments with the time to implement the sustained WASH solutions and strengthening of health systems that are necessary for long-term cholera control. In this way, it provides a solution to the political challenges around taking action on cholera, as it demonstrates that recurring cholera outbreaks are not an irresolvable condition even in the most resource-poor environments. Ongoing efforts to ensure OCV supply will be critical in coming years, because increasing government interest in cholera control is likely to cause the demand for OCVs to grow faster than the supply.

This demand is reflective of the fourth and most important enabling factor—an increasing degree of political commitment from key countries to reducing or eliminating the burden of cholera within their borders. The rise in interest and commitment comes, in part, from an increasing recognition that preventing cholera is not just affordable but also has a high return on investment. The costs of responding repeatedly to outbreaks in a reactive manner are higher than the costs of proactively addressing cholera control using a development approach. Efforts are underway to better quantify the economic burden of cholera in endemic countries. An analysis was recently undertaken of 14 cholera-affected countries in Asia, which were estimated to have a total of 850000 cholera cases and 25500 deaths in 2015 (V. Mogasale, V. V. Mogasale, and A. Hsiao, unpublished data). The investigators estimated that in these countries in 2015, cholera cost $20.2 million in out-of-pocket expenses, $8.5 million in public sector costs, and $12.1 million in lost productivity due to illness. Lost productivity due to premature deaths was estimated to be $985.7 million. Given that Asia represents about 39% of estimated global cholera cases, this estimate reflects only part of the global economic burden of cholera.

A case study on the Democratic Republic of Congo developed for the *Global Roadmap* showed that the successful implementation of cholera control measures in alignment with the *Global Roadmap* may allow up to 50% cost savings compared with the ongoing average yearly cost of continuously responding to emerging cholera outbreaks [2]. Most importantly, this approach also significantly reduces the impact of all water-related diseases, putting countries in the best position to achieve their national health and development targets, as well as the SDGs.

Governments and development donors have often taken the view that WASH infrastructure is simply too expensive to implement in the near term in cholera-affected areas. Yet we know that access to basic safe water and on-site, nonsewered sanitation is sufficient to prevent the vast majority of cases of cholera because the disease is usually transmitted in heavily contaminated water. Providing access to basic WASH services may cost as little as $40 per person in capital costs covering a 10-year period, and, in addition to preventing cholera, prevents a wide range of other water-related illnesses, as well as contributing to achieving goals related to poverty, malnutrition, and education.

Although the global health community has largely shifted its approach away from disease-specific efforts and toward systems strengthening, cholera control finds itself in line with this approach, given its emphasis on strengthening laboratory capacity, disease surveillance, and health system preparedness. Multisectoral cholera control plans also serve as a useful mechanism for bringing health actors and WASH actors to a single table, forging lines of communication and coordination and developing relationships that are valuable beyond cholera control.

Finally, the GTFCC offers a strong platform for implementation of the *Global Roadmap.* Since its revitalization was completed in 2014, the GTFCC has become a highly energized, well-resourced network of organizations that are individually and collectively intensifying their efforts to control cholera at all levels. In October 2017, the partners launched the renewed global cholera control strategy (the *Global Roadmap*) and agreed at the leadership level to commit their resources to supporting countries’ efforts to reduce cholera deaths by 90% by 2030. The *Global Roadmap* provides an effective mechanism for synchronizing the efforts of countries, donors, and technical partners, allowing for a coordinated, multisectoral approach to country-level planning for cholera control.

Every case and every death from cholera is preventable with the tools we have today, putting the goal of ending cholera within reach. If political commitment to ending cholera remains strong, we are likely by 2030 to live in a world with 90% fewer cholera deaths, in which the world’s poorest persons have a renewed opportunity for health and development.

## Notes

***Acknowledgement.*** Thanks to the partners of the Global Task Force on Cholera Control for their engagement in support of the 2030 Roadmap.

***Supplement sponsorship.*** This work is part of a supplement sponsored by the Bill and Melinda Gates Foundation.

***Potential conflicts of interest.*** All authors: No conflicts of interest.

## References

[1] Ali M, Nelson AR, Lopez AL, Sack DA Updated global burden of cholera in endemic countries. PLoS Negl Trop Dis 2015; 9:e0003832. 26043000 10.1371/journal.pntd.0003832 PMC4455997

[2] Global Task Force on Cholera Control. Ending cholera: a global roadmap to 2030. 2017 http://www.who.int/cholera/publications/global-roadmap.pdf?ua=1. Accessed 15 March 2018.

[3] World Health Organization estimates, 2017.

[4] Lessler J, et al Mapping the burden of cholera in sub-Saharan Africa and implications for control: an analysis of data across geographical scales. Lancet 2018; 391:1908–15 29502905 10.1016/S0140-6736(17)33050-7 PMC5946088

[5] World Health Organization/UNICEF. Progress on drinking water, sanitation and hygiene: Progress on drinking water, sanitation and hygiene: 2017 update and SDG baselines http://apps.who.int/iris/bitstream/10665/258617/1/9789241512893-eng.pdf?ua=1 Accessed 20 August 2018.

[6] World Health Organization. Diarrhoeal disease. WHO fact sheet. Updated May 2017 http://www.who.int/mediacentre/factsheets/fs330/en/ Accessed 20 August 2018.

[7] Deployments from the oral cholera vaccine stockpile, 2013–2017. Wkly Epidemiol Rec 2017; 92:437–42. 28799734

