# Corrigendum

**DOI:** 10.1093/infdis/jiy619

**Published:** 2018-11-15

Corrigendum to Global Cholera Epidemiology: Opportunities to Reduce the Burden of Cholera by 2030 by Dominique Legros. *The Journal of Infectious Diseases*, jiy486, https://doi.org/10.1093/infdis/jiy486.

The text “on behalf of the partners of the Global Task Force on Cholera Control” has been removed from the list of authors, and the following Acknowledgement has been added: “*Acknowledgement* – Thanks to the partners of the Global Task Force on Cholera Control for their engagement in support of the 2030 Roadmap.”

